# Assessing Patient Safety Culture in Hospital Settings

**DOI:** 10.3390/ijerph18052466

**Published:** 2021-03-03

**Authors:** Abdulmajeed Azyabi, Waldemar Karwowski, Mohammad Reza Davahli

**Affiliations:** Department of Industrial Engineering and Management Systems, University of Central Florida, Orlando, FL 32816, USA; wkar@ucf.edu

**Keywords:** patient safety culture, safety climate, behavioral learning, healthcare

## Abstract

The current knowledge about patient safety culture (PSC) in the healthcare industry, as well as the research tools that have been used to evaluate PSC in hospitals, is limited. Such a limitation may hamper current efforts to improve patient safety worldwide. This study provides a systematic review of published research on the perception of PSC in hospitals. The research methods used to survey and evaluate PSC in healthcare settings are also explored. A list of academic databases was searched from 2006 to 2020 to form a comprehensive view of PSC’s current applications. The following research instruments have been applied in the past to assess PSC: the Hospital Survey on Patient Safety Culture (HSPSC), the Safety Attitudes Questionnaire (SAQ), the Patient Safety Climate in Health Care Organizations (PSCHO), the Modified Stanford Instrument (MSI-2006), and the Scottish Hospital Safety Questionnaire (SHSQ). Some of the most critical factors that impact the PSC are teamwork and organizational and behavioral learning. Reporting errors and safety awareness, gender and demographics, work experience, and staffing levels have also been identified as essential factors. Therefore, these factors will need to be considered in future work to improve PSC. Finally, the results reveal strong evidence of growing interest among individuals in the healthcare industry to assess hospitals’ general patient safety culture.

## 1. Introduction

According to the World Health Organization, patient safety (PS) is about preventing medical errors and their adverse effects on patients during healthcare delivery [1,2,3]. Unsafe medical practices can lead to patient injury, death, or disability [4]. The proliferation of such incidents has led to the recognition of the need to improve patient safety culture (PSC) in the healthcare industry worldwide. Furthermore, patient safety has been considered as one of the strategic components of healthcare management [5]. Kohn et al. [6] argued that safety is a crucial and fundamental aspect of patient care research. Kohn et al. [6], in a landmark of PS publications, advocate for error prevention and mitigation using a systematic approach to PS management. Therefore, to ensure the highest level of safety culture in the healthcare industry, it is also essential to understand the beliefs, attitudes, norms, and values of PS and its thresholds [7].

The present study focuses on patient safety culture (PSC) in hospitals. This article’s main objective is to discuss the research tools used to assess PSC and identify its essential components. The preferred reporting items for systematic reviews and meta-analyses (PRISMA) were used for this review to ensure reliable results. The PRISMA protocol contains 27 items that aim to analyze and report scientific evidence reliably [8].

This paper is structured as follows: the methodology section explains research questions and research strategy; the results section represents the primary outcomes; the discussion section answers research questions.

## 2. Materials and Methods

This review aimed to evaluate current research on PSC in the healthcare setting. The following two research questions have been posed:

1. What research instruments are used to study patient safety culture?
2. What are the essential dimensions of patient safety culture assessment?

The study follows the guidelines of PRISMA, as discussed by Moher et al. [8]. First, the protocol was used to specify the search strategy and research questions. Next, the Hawker Assessment Tool was used to assess the quality of the articles identified [9]. Sources for the systematic review included peer-reviewed articles, proceedings, textbooks, conference presentations, and reference books within the scope of PSC. At the exploration stage, the bibliography search focused on academic databases, including CINAHL, MEDLINE, Embase, ProQuest, Google Scholar, PsycINFO, and PubMed. Each of these databases provided adequate information regarding PSC in hospitals.

Eligibility criteria for the search space were applied to articles published after 2006. Articles were identified based on the combination of keywords 1-4, as illustrated in Table 1.

The eligibility criteria allowed us to narrow down the subject literature and to identify publications that were relevant to the stated research questions. The articles selected for this study met specific inclusion criteria; namely, these papers (a) were written in English; (b) had been peer-reviewed; (c) identified or described PSC; (d) applied to hospital settings; (e) utilized a survey tool to measure dimensions of PSC among acute care hospital personnel; and (f) applied to general, secondary, tertiary, teaching, or university hospitals. Exclusion criteria included (a) book chapters; (b) papers that, upon review, were found to not be related to the research questions; (c) opinions, viewpoints, anecdotes, letters, and editorials; (d) studies with small sample sizes; and (e) case studies that focused on only one specific hospital unit or sector. Paper titles and abstracts were analyzed based on the stated inclusion and exclusion criteria. Any discrepancies that arose during this phase were resolved through a process of discussion and consensus.

Hawker et al. [9] noted that the quality of any given paper must be assessed against a set of predefined criteria to determine whether it is appropriate for further study. They also proposed that such an appraisal should be performed through the use of appropriate appraising tools. The present study applied the Hawker Assessment Tool, which enables the user to score the quality of papers reviewed. This tool has a uniform assessment form for all types of papers, thereby providing consistency in the evaluation process. One of the assessment factors is the consideration of whether the abstract offers a description of the study. Other factors include the introduction of the paper under review, the paper’s aims, background study, and findings. This tool also enables the user to analyze the study’s implications concerning the topic under review and indicates how the findings can be converted into policies. A maximum score of 36 [9] was used to assess the quality of potential papers to be included in the present study. The range of the reviewed studies’ quality score ranges from a minimum of 9 points to a maximum of 36 points. To create the overall quality grades, we used the following definitions: high quality (A), 30–36 points; medium quality (B), 24–29 points; and low quality (C), 9–24 points.

A data extraction template from the Hawker Assessment Tool was used to collect data regarding the properties of the adopted studies. This template allows for a literature analysis with a minimal selection bias [10,11].

**Figure 1:**
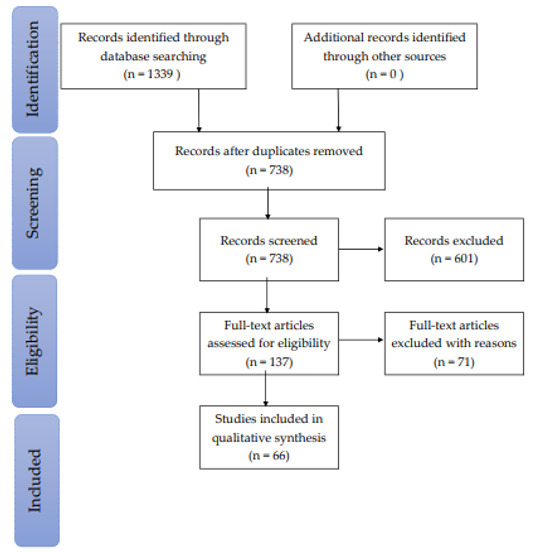
Flow diagram of the methodology and selection process [8].

Through a search of all relevant databases, a total of 1339 publications were initially identified. The databases searched included CINAHL, MEDLINE, Embase, ProQuest, Google Scholar, PsycINFO, and PubMed. Further analysis was required to eliminate duplicate titles, which resulted in 601 duplicates being discarded. This step was followed by the application of exclusion criteria, as previously described. The abstracts for the remaining 261 titles were read, which led to the selection of 137 relevant articles whose entire texts were analyzed. It should be noted that no additional articles were added after the references from the initially selected papers were examined. Figure 1 provides a flowchart illustrating the article selection process. A total of 66 articles that met all eligibility criteria and that had been published between 2006 and 2020 were selected for the study.

To identify research instruments used to study patient safety culture, two researchers (authors) independently read the selected articles’ full texts to identify research instruments and their aspects. Subsequently, the two authors compared their findings to develop unified results. Disagreements between the two researchers concerning research instruments and their identified aspects were discussed and resolved in sessions with the third researcher.

## 3. Results

All included records were categorized according to objective, strength, limitation, finding and quality score as it is represented in Table 2.

**Figure 2:**
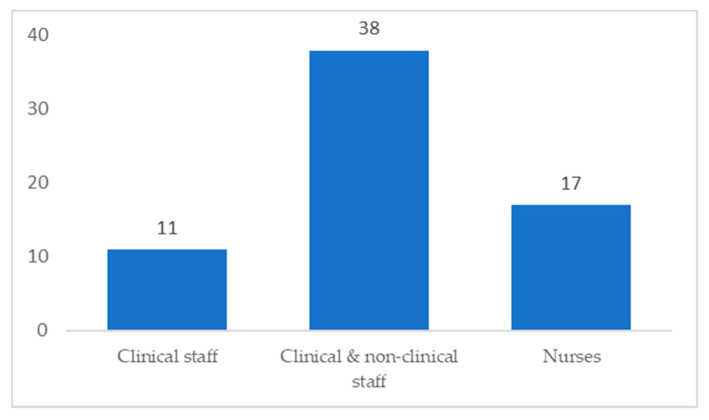
Focus of each study according to participants.

A total of 1,690,225 participants took part in the reviewed studies. The response rate ranged from 17% [53,58] to 100% [39]. However, some studies did not report the response rate [15,28,34,45,46,67]. The study participants included nurses, doctors, and administrators. Figure 2 shows the distribution of participants. Seventeen papers focused on nurses, 38 studies included clinical and non-clinical staff, and 11 studies included clinical staff only.

The reviewed articles reported several limitations concerning the applied methodology and results. First, articles mainly used quantitative approaches to measure PSC, where these methods are not efficient for measuring complex and dynamic attributes such as culture. Second, cross-sectional designs were commonly used among included articles with data collected at one point at a time. Therefore, it is not possible to determine the causal relationships between PSC and the explanatory variables. Third, self-reported questionnaires were applied to collect data, which introduced social desirability biases to the reported research results. Fourth, seven articles did not report their participants’ response rate, and 26 articles reported a relatively low response rate (less than 60%). The majority of the reviewed papers concluded that their results could not be generalized because their studies represented unique cultures, the large variations of the applied research instruments, variation in sample sizes, differences in the type of healthcare facilities, and the diversity of study participants.

**Figure 3:**
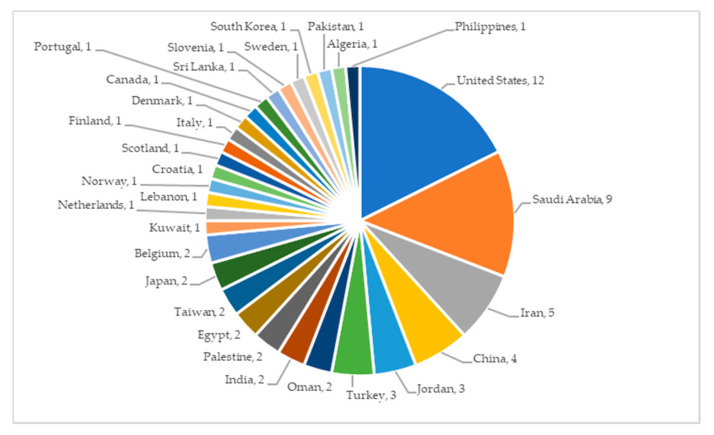
Global distribution of the articles included in this analysis.

The global distribution of the included articles is represented in Figure 3. Several studies targeted more than one country.

**Figure 4:**
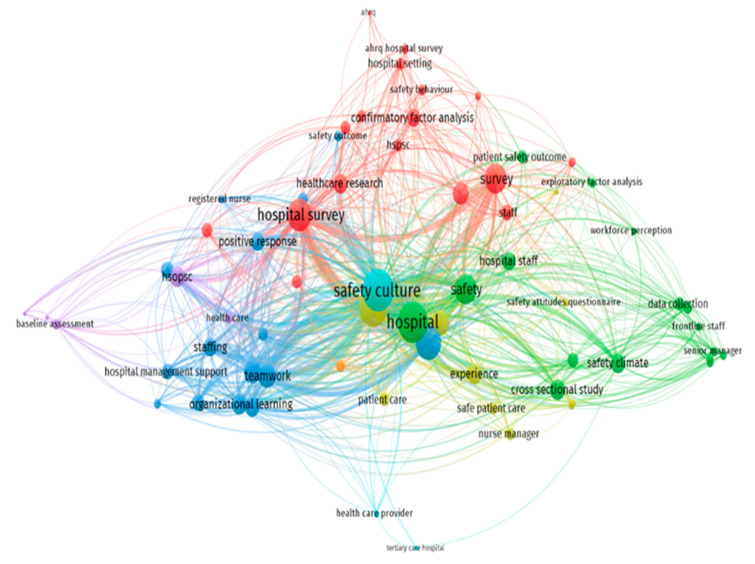
The map of the co-occurrence of terms in the title and abstract.

**Figure 5:**
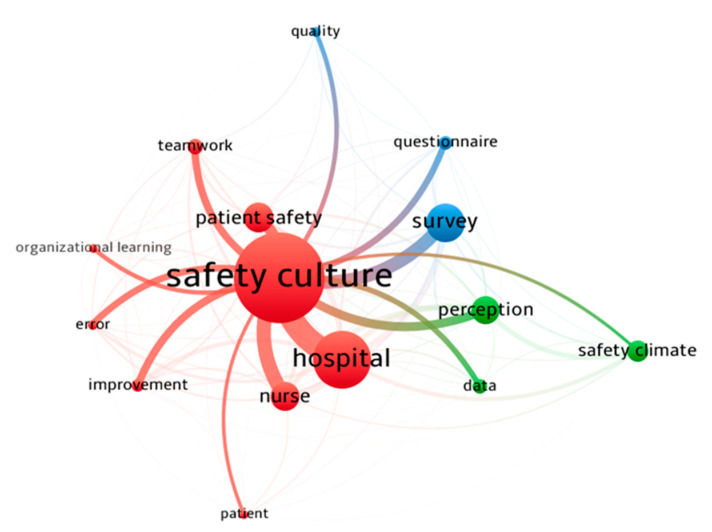
The map of the co-occurrence between safety culture and other high-frequency terms.

The map of the co-occurrence of terms in included papers is depicted in Figure 4. The nodes represent specific terms, their sizes indicate their frequency, and links show the co-occurrence of the terms. In the title and abstract of included papers, frequently co-occurring terms created a cluster that appeared with the same color (green, blue, and red color). The three core nodes of these clusters are safety climate, safety culture, and survey. Furthermore, the relationship between the core node of “safety culture” and other high-frequency terms is shown in Figure 5. The thickness of links between nodes represents the strength of the co-occurrence relationships.

## 4. Discussion

In this section, two research questions are answered in two subsections of PSC instruments and PSC dimensions.

### 4.1. PSC Instruments

This review identified five primary instruments that have been used to assess PSC in hospital settings. The first instrument, the Hospital Survey on Patient Safety Culture (HSPSC), was used in 54 studies. By contrast, the Safety Attitudes Questionnaire (SAQ) tool was used in five studies, and the Patient Safety Climate in Health Care Organizations (PSCHO) was used in five studies. The Scottish Hospital Safety Questionnaire (SHSQ) and the Modified Stanford Instrument-2006 (MSI-2006) were used by one study each as shown in Table 3.

#### 4.1.1. Hospital Survey on Patient Safety Culture (HSPSC)

In 2004, the AHRQ developed the HSPSC within the United States (U.S.) Department of Health and Human Services, which became a widely used survey. This survey allows for an assessment of staff opinions concerning medical errors, adverse event reporting, and other issues relevant to PS [12,13]. Although the original survey was primarily intended for use by hospitals, it has been enhanced with various versions. This survey currently measures the safety culture of patients in ambulatory settings, outpatient health offices (such as primary care), nursing homes, and public pharmacies. The HSPSC is available in different languages, including Arabic, Spanish, French, and Dutch. The hospital questionnaire version contains 42 items and assesses 12 composites that are treated as subscales.

#### 4.1.2. Safety Attitudes Questionnaire (SAQ)

The SAQ was developed by Sexton and colleagues at the University of Texas in the U.S. The SAQ comprises six main components (Table 3). The primary advantage of the SAQ is that it can be applied to different healthcare settings. The complete version of the SAQ uses a total of 60 components or items, with 30 items considered as standard across all environments. The survey utilizes a five-point Likert scale ranging from strongly agree to strongly disagree. In addition to the 30 standard items, this survey can incorporate another 6 items, with 3 additional items that focus on demographic studies. The statements utilized by the short-form SAQ can also be addressed using the five-point Likert scale. The short form is easily accessible and available in different languages, including English, Swedish, Dutch, Norwegian, German, Arabic, and Chinese [73].

#### 4.1.3. Patient Safety Climate in Health Care Organizations (PSCHO)

According to Singer et al. [74], PSCHO was designed with the aid of the Stanford Safety Instrument. The PSCHO tool includes 38 items that are used to assess work units, interpersonal factors, and inter-related organizational topics [74]. Using a Likert scale, items are rated via a two-page form. PSCHO is considered to be the first tool that analyzed safety constituents and provided information by measuring the safety climate in corporations outside hospitals. Information from this survey regarding management and clinical personnel can be applied to a wide range of healthcare organizations. PSCHO has undergone psychometrical tests and can be used to compare the performance of several types of hospital units. The earlier form of this tool has been modified with respect to its length [75] and has been adapted for use in multiple languages [61].

#### 4.1.4. Scottish Hospital Safety Questionnaire (SHSQ)

The SHSQ was designed for the Scottish NHS clinical staff, with the main aim of gauging the safety outcomes and climate for both patients and staff. The SHSQ includes 4 primary components: 44 items related to the hospital survey (HSPSC), 10 worker safety behavior aspects, 2 items concerned with self-reported patient and worker injuries (see Table 3), and 7 items that focus on demographics [54].

#### 4.1.5. Modified Stanford Instrument-2006

The MSI-2006 Patient Safety Culture in Healthcare Organizations Survey [53] was designed to evaluate 32 unique items encompassing five aspects. These aspects include, but are not limited to, issues associated with seeking help, shame, and self-awareness (Table 3). Modification of the MSI-2006 tool has facilitated the assessment of perceptions of a wide range of hospital staff, including direct care workers, technicians, health practitioners, managers, and nurses. This tool also includes assessments of other aspects, such as support service personnel, as these workers are an essential part of the hospital and healthcare setting. MSI-2006 was developed for a wide range of hospital settings with the aim of generating relevant and accurate data over the long term.

### 4.2. PSC Dimensions

To understand the effect of PS on healthcare organizations and their staff, the process and structure of each system needs to be broken into subsystems. The type of instruments and their varying dimensions, as well as the groups targeted in each study, were among the most interesting points to be considered when attempting to understand PS.

Five instruments were used in the reviewed studies to measure PSC within the healthcare facilities examined. As indicated in Table 2, teamwork, organizational and behavioral learning, reporting of errors and safety awareness, gender and demographics, work experience, and staffing levels were perceived as factors that significantly impacted patient safety. Personal variables, such as the age and experience of medical professionals, were also related to PS perceptions. By examining results from individual hospitals or groups of hospitals, we identified the aspects of safety culture that need improvement, including considerations of working conditions and management support.

The reviewed studies differ in their focus on relevant PS variables across different hospitals in various geographical regions. However, many standard components of safety culture indicators and risk factors have been identified [14,15,18].

#### 4.2.1. Teamwork

Teamwork and mutual help provided by team members in task performance within specific hospital units were the factors that represented PS through the use of different instruments [77]. A high score of positive teamwork within units indicates the existence of healthy work relationships and respect among members within a unit [67]. Moreover, vertical hierarchy, horizontal hierarchy, and years of working within a unit influenced the level of teamwork within units. The level of skill competency also affected teamwork within units [57]. However, teamwork across units was reported to have low positive scores [15,21]. Besides, attitudes towards colleagues from different units and managers’ or supervisors’ actions and expectations towards PS affected teamwork performance across units [18]. According to Hamdan and Saleem [19], skills and organizational learning were significantly related to knowledge teamwork across units. However, supportive managers or supervisors increased the level of teamwork across units. Moreover, colleagues who worked closely together and supported each other in their work duties often resulted in mutual respect [19]. Therefore, while it could be concluded that teamwork is one of the important factors that impact PS, there are always opportunities for improvement.

After reviewing the studies, the HSPSC and SAQ instruments are the only two that are focused on the teamwork dimension. Among the studies that used the SAQ, the pronounced difference in PSC was notable among the front-line healthcare staff, supervisors, and managers [65]. Furthermore, a great variance in PS perception was observed within specific hospital units compared with differences between units. Chakravarty et al. [39] reported low variations in scores between hospitals based on the PS index. However, their study also revealed significant differences in individual measures of PS, including the perception of management, teamwork, and stress recognition, when using the PS index score [39].

The HSPSC provides more details about teamwork performance within and between units of hospitals. Additionally, teamwork is the most factor that has a relationship with the other characteristics of PS. Among studies using the HSPSC, high scores were obtained for teamwork within units, especially in different developing countries [18,30,35,43,45,47,56,67]. These results confirm that the healthcare industry greatly relies on interdisciplinary teams of specialists with the skill sets needed to perform specialized tasks. Such teams also collaborate to achieve common safety goals [40]. Different teams use shared resources and rely on communication to adapt to ever-changing healthcare environments. The behavior of these teams was analyzed through observational studies. The results indicated that the teams’ clinical performance was influenced by how they communicated, coordinated, and practiced effective leadership [40].

#### 4.2.2. Organizational and Behavioral Learning

Organizational learning is also a critical factor that affects the PS. In most of the survey studies examined, positive responses were given for organizational learning/continuous improvement as a composite for PS [12,29,31,34]. Continuous improvement can be gained from daily work routines and incidents. PS can also improve by enhancing relevant personnel’s skills and knowledge based on incident analysis. Additionally, the junior staff can learn from more experienced staff as they worked together [74].

Although organizational and behavioral learning had positive responses, the outcome dimension, frequency of events reported, did not have positive responses in all the studies included in this review. Therefore, the learning process in PSC should be enhanced by establishing formal methods instead of informal practices to avoid harming patients. In the U.S., as a result of the IOM’s report, the U.S. Congress passed the Patient Safety and Quality Improvement Act in 2005, which aimed to improve quality and safety via the collection and analysis of data on patient events. This shows that PS has to be enhanced by the participation of healthcare providers and patients.

In 28 of the studies examined, 55% of the participants agreed that these factors were important components of organizational learning and continuous improvement processes at the examined healthcare facilities. These processes are also responsible for communicating and conveying information that is essential for PS and healthcare. Such processes occur in both formal and informal learning environments within healthcare systems that perform complex and interconnected operations, which should be considered to enhance the PSC.

#### 4.2.3. Reporting of Errors and Safety Awareness

Two of the dimensions that received low positive scores were non-punitive responses to errors and the frequency of event reporting [32]. That is because a large percentage of respondents indicated that they do not report incidents to their managers or supervisors. The reason behind this could be that staff members fear being reprimanded for an error and the lack of safety awareness. Such a culture might cause the staff to hide issues that could later influence the efficacy of PS. A culture that includes non-punitive responses to errors could arise from managers, supervisors, and colleagues [46]. Another reason behind this finding could be the risks of patients complaining; patient demands for compensation might have also reduced the frequency of event reporting [52].

Moreover, another study that was conducted in Saudi Arabia illustrated that one of the dimensions that indicated a high positive response was feedback and communication about errors [24]. The factors requiring improvement included non-punitive responses to error reporting and adequate personnel staffing [24]. The survey showed that the overall perception of PS was 59.9%, while the reporting frequency was 68.8% [24]. Another study that was conducted in Scotland by Agnew et al. [54] found that the overall perception of PS was judged at 56%; the reported frequency of incident reporting was also 56%. Another study in Saudi Arabia showed that the frequency of reporting adverse safety events was 57% [23]. Additionally, A study conducted by Khater et al. [26] among senior nurses in Jordan showed a positive correlation between non-punitive responses to medical errors and the frequency of medical error reporting. The result was a reduction in adverse events regarding PS and risks of complaints from patients. The overall perception of senior nurses was 51.5% before education and 60.6% after educational sessions. The frequency of event reporting increased from 54.2% to 64.3% after implementing suitable educational training [26].

In a related study, Hellings et al. [49] described a PSC improvement approach implemented in five Belgian hospitals. The results showed that management support for PS increased along with supervisor expectations and actions that promoted safety practices. Medical personnel from Dutch-speaking hospitals had a higher positive perception of PS compared with French-speaking hospitals [49]. The survey also showed that respondents working in pediatrics, rehabilitation, and psychiatry departments (units) provided more positive feedback about perceived PSC. By contrast, medical professionals working in emergency departments (units) provided lower positive feedback [49]. These differences in the hospitals’ departments and languages are some of the reasons for reporting low scores in the non-punitive responses to errors [49].

A positive perception of PS was observed among medical personnel in China and U.S. managers. In both countries, these individuals expressed a higher level of perceived PS compared with front-line personnel. However, Chinese staff had higher scores for work-related fear of shame and blame compared with their American counterparts [61]. The U.S. hospitals have fewer cases of “fear of blame” compared to Chinese hospitals [61].

As noted earlier, a reduction in avoidable incidents with potential or actual medical harm is a key objective in developing a robust PSC [31,34,36]. Harm can be measured by the frequency of reported events. Effective reporting of safety incidents is essential for identifying the causes of failures in a healthcare work environment. The present analysis indicates a need to implement more effective reporting systems. Reporting provides relevant information about the frequency of events that can adversely affect PS.

A culture of blame was evident in 22 studies, representing 43% of those examined. In these studies, punitive responses to medical errors were prevalent and created a culture that discouraged personnel from reporting safety incidents and occurrences [42]. Such a culture impeded the hospitals’ ability to determine the causes of errors and, consequently, to learn from previous mistakes [13,15,17]. In instances in which an influential safety culture exists, workers can highlight potential risk factors and also identify failures when they occur with a focus on PS [38]. Additionally, adverse events arise from multiple unintentional causes. Blame was judged to be appropriate when addressing individuals who consistently commit frequent and careless errors or who ignore established safety standards and policies. Competent institutions should maintain a culture of accountability to ensure that patient care is maintained at the highest levels.

A study conducted in Canada by Zaheer et al. [53] focused on supervisory and senior leadership support for PS. The survey noted that ease in reporting provided the hospital with a platform for learning and improving through reported incidents. Among the supervisory and senior leadership, ease in reporting was recorded at 11% and 12%, respectively. These findings suggest that hospitals should ensure that front-line staff are aware of and contribute to their organization’s reporting systems. Ease in reporting should provide organizations with an opportunity to improve strategy, commitment, and the overall efficacy of PSC in sample facilities [53].

#### 4.2.4. Gender and Demographics

PSC is a multidimensional concept that requires a strict analysis to identify its vital elements. The perception of PSC is always measured through the dimensions of the tools used. However, gender and demographic characteristics can be used to analyze participants’ responses to a survey [16]. Many of the studies analyzed herein demonstrate the correlation between PSC perception with gender and demographics.

Numerous differences in nurses’ perceptions of PSC arose due to demographic characteristics, including gender, age, level of education, years of experience, the language used, and length of work shift [27]. In general, female nurses had a more positive view of the prevalent PSC than did their male counterparts. Moreover, nurses between the ages of 40 and 60 years had a more positive view of the PSC than nurses between 20 and 40 years of age [53]. As 85.4% of the nurses had a Bachelor of Science in nursing, it is plausible that their education levels did not affect their perception of PS [16]. However, as Hamdan et al. [19] observed, education is generally one of the most critical aspects of healthcare delivery to patients worldwide.

Elsous et al. [27] evaluated nurses’ perception regarding PSC and investigated the influence of age, hierarchal position, working hours, and experience. Job satisfaction and perception by management concerning PS had a strong influence on these variables. Front-line clinicians had a less positive attitude toward PS than did nurse managers. Moreover, positive attitudes increased with years of experience. Work shift hours and ages of the nurses had a direct effect on the perception of PS. Nurses working within the normal hours allocated per week and aged 35 years or older showed a better PS perception [27]. The study also reported no differences in safety attitude scores between nurses and doctors due to gender, age, and work experience [27]. The studies of the potential effects of gender and demographics on the perception of PSC should be expanded in the future.

#### 4.2.5. Work Experience

Relevant work experience was strongly related to the perception of the PSC. Work experience was also associated with the perceived quality of care among nurses [19]. Furthermore, more experienced healthcare providers had a better understanding of patient care needs than did less experienced nurses [53]. A study conducted in the U.S. by Hansen et al. [76] investigated the relationship between hospital PSC and rehospitalization rates within 30 days of discharge. A survey done in 67 hospitals discovered that higher readmission rates of acute myocardial infarction and heart failure patients were directly related to a lower safety climate [76]. Additionally, frontline staff workers reported a lower level of perceived safety climate with the readmissions, which were the management’s responsibility [76]. In another study, a survey was conducted in 97 hospitals in the U.S. that revealed that frontline workers perceived a climate of safety more frequently than did the managers and the supervisors [77]. Furthermore, among the clinicians, nurses perceived a safety climate more than physicians [77]. Based on that, it could be concluded that the work environment plays a key role in perceiving the PSC.

Moreover, another study illustrated that language also has effects on perceiving the PSC [16]. Non-Arabic-speaking nurses had more positive views of PSC than did Arabic-speaking nurses [16]. This finding was unanticipated as the Arabic-speaking nurses and their patients spoke the same language. The low PSC scores might have been due to disparities in educational systems affecting PS perceptions. Furthermore, nurses working on day shifts had more positive PSC perceptions than nurses working night shifts or alternating shifts [16]. It was noted that day-shift nurses were more time engaging with and involved in their patients’ progress, which resulted in a positive PSC [16]. Day-shift nurses also interacted with their managers and became more familiar with relevant aspects of the PSC [16]. Therefore, it could be concluded that work experience and the possibility of knowledge exchange had a measurable impact on perceptions related to the PSC.

#### 4.2.6. Staffing

The availability of human resources also impacts the perceptions of the PSC. A study conducted in Scotland by Agnew et al. [54] aimed to analyze the relationship between the medical personnel safety behavior and reported injury measures for patients and healthcare providers. At the hospital level, the authors found a strong correlation between overall PS scores and patient and personnel injury measures and behavior [54]. Therefore, the level of hospital staffing, coupled with management support for PS, also influenced the perception of PS within the studied facilities [54]. Generic safety climate factors and patient-specific items showed a strong correlation with perceived safety outcomes [54]. To summarize, a total of 24 studies reported on the issue of healthcare personnel understaffing. The staff reported feelings of being overburdened and overloaded with their daily responsibilities in approximately half of the hospitals [18,37,47,48,60,64,66]. Consequently, this issue had a negative impact on the quality of care provided by the staff [45]. Therefore, the availability of adequate staffing plays a critical role in perceiving the PSC because employees’ focuses might be harmed due to overload.

## 5. Study Limitations

The present study has some important limitations. This systematic review focused only on articles written in English; moreover, a meta-analysis was not performed. The results of the reviewed studies are difficult to generalize due to the application of a diverse set of PSC measures with different dimensions. Furthermore, the reviewed studies also varied in the type of participants included (doctors, nurses, and administrators), the periods over which the measurements were conducted, the sampling strategies used, and the cultural settings. For example, the results that focused primarily on results from nurses were obtained from convenience samples of participants and as such cannot be generalized to the entire nursing staff. Finally, this study did not account for language and cultural disparities prevalent in the specific countries in which the reported studies were conducted.

## 6. Conclusions

Enhancing the perception of the PSC in health sectors plays a key role in improving their overall quality, efficiency, and productivity. This paper contributes to the body of knowledge related to PSCs by identifying important critical factors and illustrating the instruments that have been developed and used to generate data. A comprehensive review of perceiving the PSC in hospital settings was provided. A systematic literature review was conducted using the PRISMA protocol for the period of 2006 to 2020. The paper reviewed 66 studies that were identified based on carefully selected keywords. The Hawker Assessment Tool was also implemented in this paper to enable the researcher to score the quality of the papers reviewed. The paper analyzed PSC perception in the hospital setting, determined available instruments, and identified the most critical factors that have an impact on the PSC. Our findings revealed that teamwork and organizational and behavioral learning are some of the factors that have a significant impact on the PSC. This paper also illustrated that reporting errors and safety awareness, gender and demographics, work experience, and staffing are additional critical factors that need to be considered further to improve perceptions of PSCs.

In the future, the impact of culture on PS might be analyzed in greater depth. PS, particularly in hospitals, is a dynamic and complex phenomenon. Therefore, it is recommended that research and surveys be performed every two to three years to ensure the best practices for PS. Such an approach could also enhance the quality of healthcare delivery. A large number of hospitals in many different countries have been studied and the specific characteristics of the healthcare management systems in these countries greatly vary. Consequently, for future studies, a broader study population crossing the national boundaries would help to ensure that the findings can have an impact on the development of high-quality, affordable healthcare worldwide.

Finally, it should be pointed out that although the reported survey questionnaires described in the reviewed studies were anonymous, some respondents might not have been candid in providing their answers. Some of the questionnaires were long and some of the respondents may have become distracted during the process, lost interest, or answered some questions inaccurately. Additionally, some inconsistencies in using different survey tools due to cultural and language diversities were noted. For future, investigations including qualitative evaluations of these relationships should be conducted. Finally, the long-term effects of safety incidents on patients’ health and their long-term impact on families have not been investigated. Future studies should evaluate the effects of such experiences in hospital settings.

## Figures and Tables

**Table 1:** Keywords used in the present review.

Row	Step
Keywords 1	“safety culture” OR “safety climate” OR “patient safety culture” OR “patient safety climate” OR “patient safety”
Keywords 2	“perception” OR “measure” OR “evaluate” or “assess” OR “survey” OR “instrument ” OR “tool”
Keywords 3	“hospital ” OR “teaching” OR “tertiary”
Keywords 4	“nurse” OR “doctor” OR “physician” OR “staff” OR “health professional”
Search	#1 AND #2 AND #3 AND #4

**Table 2:** Publications included in the literature review.

Instrument/Year/Country/Reference	Aim(s)	Strength(s)	Limitation(s)	Finding(s)	Quality Score
**HSPSC, 2019, Saudi Arabia [12]**	Investigate the perceptions of healthcare professionals toward PSC in hospitals throughout the Hail region	Variety of healthcare professionals (nurses, physicians, and administrators/managers) considered for collecting data. Response rate among participants was 99.22%	Only four hospitals considered for data collection.	Healthcare professionals have a positive perception of patient safety. Organizational learning was the strongest area in PSC. Professionals with a greater number of employment years were more willing to communicate. Among respondents, 63.53% stated that they had never reported a case of patient safety. The low rate of reported cases was attributed to fear of the cases being recorded in the respondent’s file.	32
**HSPSC, 2012, Saudi Arabia, [13]**	Identify general strengths and recognize areas of patient safety improvements	Variety of clinical and medical staff (physicians, nurses, technicians, pharmacists, and others) considered.	Response rate among participants was 61%. Only two general hospitals considered	Organizational learning/continuous improvement and teamwork within units received positive outcomes at 79% and 77%, respectively. Non-punitive responses to errors and staffing had low positive response rates at 22% and 31%, respectively, representing areas for improvement. The overall percentage of positive responses among dimensions of patient safety was 58%.	27
**HSPSC, 2016, Turkey, [14]**	Explore and describe nurses’ perceptions of PSC	Response rate among participants 74%. HSPSC Turkish version used.	Only nurses in four hospitals (one university hospital and three general hospitals), and nurses consiered for collecting data	The mean positive response rate for the 12 PSC dimensions of the HSPSC survey was 52%. Within units and organizational learning/continuous improvement were reported. Non-punitive responses to errors and frequency of event reporting were areas for improvement. Nurses who had worked for more than 10 years in their profession showed significantly higher PSC scores in all dimensions. Nurses working in ICUs had higher scores than those working in other units in all patient safety dimensions. 50.2% of the nurses rated the level of patient safety as good or excellent. Among nurses, 80.4% indicated that they had never reported an error. The overall perception of patient safety was 51%.	27
**HSPSC, 2012, Egypt, [15]**	Assess PSC perceptions among healthcare providers and identify factors that may critically affect PSC	Variety of healthcare professionals (doctors, nurses, and technicians) considered HSPSC Arabic version used.	No response rate reported	An average of 52% was attained for positive responses for the 12 PSC dimensions of the HSPSC survey. Non-punitive responses to errors had 24.2% while frequency of event reporting and staffing were 28.4% and 38.4%, respectively. Poor teamwork across units was identified as having a low response of 48.8%. Areas for improvement included organizational learning, handoffs and transitions, communication, and support from management. Patients started reporting errors after being educated, demonstrating the accusatory culture.	27
**HSPSC, 2013, Saudi Arabia, [16]**	Identify factors that nurses perceive as contributing to the PSC	Response rate was 83%.	Only Nurses in one Tertiary care hospital considered for collecting data.	Continuous organization learning and management support formed the best areas for the support of patient safety. Other variables such as reporting errors, staffing, and communication required improvement for better patient safety. Respondent variables such as gender, level of education, age, years of experience, length of shifts, and Arabic versus non-Arabic language created a variance in patient safety consideration. Among the nurses interviewed, patient safety was rated as good or excellent.	28
**HSPSC, 2012, Egypt, [17]**	Assess healthcare providers’ perceptions of PSC within the organization and determine factors that play a role in PSC	Variety of healthcare professionals (physicians, nurses, pharmacists, technicians, and staff) considered. Response rate was 69.1% HSPSC Arabic version used.		Dimensions with the highest scores included continuous learning and teamwork, reported at 78.2% and 58.1%, respectively. Non-punitive responses to errors had the lowest score of 19.5%, representing a dimension that requires improvement. Adverse event reporting and recording was reported at 33.4%. The hospital is a training institution, exhibiting a bias for continuous learning and low error reporting, as errors are recorded in files.	29
**HSPSC, 2013, Iran, [18]**	Assess the PSC at Islamic Azad University hospitals	Variety of clinical and diagnostic staff (physicians, nurses, midwives, assistants, staff, and radiologists) considered. Response rate was 87.5%. HSPSC Persian version used.		Teamwork within units scored 48% while non-punitive error responses scored 12%. Areas identified for improvement included staffing and non-punitive responses to errors. Among respondents, 35% had a positive view of patient safety.	24
**HSPSC, 2013. Palestine, [19]**	Assess the prevalent PSC in Palestinian public hospitals	Variety of clinical and non-clinical hospital staff (physicians, nurses, paramedical and support services, hospital managers, and supervisors) considered. HSPSC Arabic version used.	Response rate was 51.2%	Dimensions with the highest scores were teamwork within units, organizational learning/continuous improvement, and supervisor/manager expectations and actions promoting patient safety at 71%, 62%, and 56%, respectively. Non-punitive response to errors, frequency of reporting, communication, management support, and staffing had low scores at 17%, 35%, 36%, 37%, and 38%, respectively. Among respondents, 53.2% had not reported any errors in the past year. General patient safety was ranked as excellent or very good by 63.5% of the respondents.	25
**HSPSC, 2010, Saudi Arabia, [20]**	Evaluate the extent to which the culture supports patient safety at Saudi hospitals	Variety of health professionals (nurses; physicians/physicians in training; pharmacists; dieticians; unit assistants/clerks/secretaries; respiratory therapists; physical, occupational, or speech therapists; technicians [lab, radiology] administration/management) in 13 general hospitals (9 public and 4 private) considered.		General patient safety was rated as very good by 60%, acceptable by 33%, and poor by 7% of the respondents. Composites that showed strength included continuous improvement, feedback, teamwork within units, and feedback and communication about errors. Staffing, under-reporting of errors, non-punitive response to errors, and teamwork across hospital units had low scores.	31
**HSPSC, 2019, Saudi Arabia, [21]**	Evaluate the PSC in Saudi hospitals and improve patient safety and quality of care by implementing safety systems and creating a culture of safety	Variety of hospital workers (physicians; nurses; pharmacists; dieticians; unit assistants/clerks/secretaries; respiratory therapists; physical, occupational, and speech therapists; technicians [e.g.,lab, radiology], administration/management) considered	Only one Tertiary hospital considered	Feedback and communication about errors had high scores, ranging from 40.7%–71.3%. Leadership, communication openness, error reporting, and teamwork across units represented areas requiring improvement.	25
**HSPSC, 2018, Kuwait, [22]**	Examine the association between the predictors and outcomes of PSC	Variety of employees (physicians, nurses, pharmacy and laboratory staff, dietary and radiology staff, supervisors, and hospital managers) in 16 public hospitals considered	Response rate was 60.5%	Continuous improvement, teamwork within units, management support for patient safety, feedback and communication about errors, and supervisor/manager expectations and actions promoting patient safety were highly scored among the respondents. General perception of patient safety was scored at 60.6% while frequency of events reported was scored at 59.0%.	33
**HSPSC, 2012, Saudi Arabia, [23]**	Perform an unbiased assessment of the impact of accreditation on PSC	Response rate was 69.5%. HSPSC Arabic version used	Only nurses in one university hospital considered for collecting data	A score of 45% was recorded for overall perceptions of patient safety. The frequency of reporting events was 57%.	30
**HSPSC, 2017, Saudi Arabia, [24]**	Reassess PSC in a large multi-site healthcare facility in Riyadh, Kingdom of Saudi Arabia, and compare it with an earlier assessment conducted in 2012, benchmarked against regional and international studies	Variety of health professionals (physicians, registered nurses, other clinical or non-clinical staff, pharmacists, laboratory technicians, dietary department staff, radiologists, and administrative staff such as managers and supervisors) considered. The results comparied with U.S.	Only one Tertiary care teaching hospital considered. Response rate was 56.7%	Teamwork within units and organizational learning/continuous improvement were strong areas while staffing and non-punitive responses to errors required improvement. A high level of correlation was observed among feedback, managerial support, organizational learning, and improved patient safety. Improvements in dimensions of patient safety from 2012 to 2015 indicated an improvement in performance. Overall perceptions of patient safety were reported at 59.5%. The frequency of reporting events was 68.8%.	33
**HSPSC, 2014, Iran, [25]**	Assess the safety culture in two educational hospitals	Response rate was 88.8%. HSPSC Persian version used.	Only nurses in two teaching hospitals considered	Non-punitive response to errors, frequency of events reported, and staffing had the lowest positive scores of patient safety dimensions. Among nurses from Afshar and Firoozgar Hospitals, 29% reported positive perceptions of patient safety.	29
**HSPSC, 2015, Jordan, [26]**	Assess PSC in Jordanian hospitals from nurses’ perspectives	Response rate was 82.2%. 21 hospitals (2 university hospitals, 4 private hospitals, and 15 governmental hospitals) considered. HSPSC Arabic version used.	Only nurses considered	A high positive response was reported for teamwork within units while teamwork across units, handoffs and transitions, communication openness, and non-punitive response to errors needed improvement. Nurses in government hospitals had lower perceptions of patient safety compared with nurses in university hospitals. Overall perceptions of patient safety were reported at 60.07%. Frequency of events reported was 69.15%.	34
**SAQ, 2017, Palestine, [27]**	Assess the perception of nurses regarding PSC and determine whether it is significantly affected by the nurses’ position, age, experience, and working hours	Response rate was 91.9%. SAQ Arabic version used	Only nurses in four public general hospitals considered	Job satisfaction and perception of management were the top variables affecting patient safety. Variables such as age, nursing position, working hours, and work experience created a variance in PSC perception. Front-line clinicians had a less positive attitude towards patients when compared with nurse managers. The longer the working experience, the higher the likelihood of having a positive attitude towards patient safety Nurses who worked the minimum weekly hours and who were 35 years or older had better attitudes towards all patient safety dimensions except for stress recognition.	33
**HSPSC, 2015, Oman, [28]**	Investigate nurses’ perceptions of PSC and identify the factors needed to develop and maintain a culture of safety among nurses		Only nurses in four governmental hospitals considered. No Response rate reported.	Feedback and communication about errors, continuous learning, and teamwork within units received high positive scores. Staffing, non-punitive response to errors, and management support attained low positive scores among the respondents. An increased number of years of experience combined with working in a teaching hospital increased the perception of PSC. The rate of positive perceptions of safety was 50.7% among respondents. Frequency of events reported stood at 58.8%.	33
**HSPSC, 2014, Oman, [29]**	Illustrate the PSC in Oman and compare the average positive response rates in PSC between Oman and the U.S., Taiwan, and Lebanon	Variety of health professionals (nurses, physicians, technicians, pharmacists, physiotherapists, and dieticians) considered. The results compared with U.S., Taiwan, and Lebanon	Only five secondary and tertiary care hospitals considered. No Response rate reported.	Organizational learning/continuous improvement had the highest positive score. Non-punitive response to errors was poorly rated among respondents. Response rates in Oman, Taiwan, the U.S., and Lebanon were similar. The overall average positive response rate was 58%. Overall perception of patient safety was 53%. Frequency of event reporting was 65%.	33
**HSPSC, 2013, Iran, [30]**	Estimate the relation between PSC and three characteristics of teaching hospitals (number of beds, education condition, and proficiency status)	Variety of staff (nurses, physicians, laboratory staff, radiology staff, midwives, operation room staff, and general managers without any specialty in therapeutic procedures) in 25 hospitals (11 teaching hospitals and 14 non-teaching hospitals) considered. Response rate was 76.8%		Highly scored dimensions included teamwork within units and organizational learning/continuous improvement. Non-punitive response to errors and staffing were the lowest positively scored dimensions. Overall perception of safety was 56.56%. Frequency of events reported was 42.85%.	29
**HSPSC, 2013, Iran, [31]**	Assess nurses’ perceptions of PSC in these hospitals	Response rate was 83.7%	Only nurses in two teaching hospital sconsidered	Organizational learning/continuous improvement had the highest positive score. Frequency of events reported, staffing, and non-punitive response to errors had the lowest scores of PSC dimensions. Overall perceptions of safety were 66.22% for the Afshar hospital and 59.5% for the Firouzgar hospital The frequency of events reported was 34.90% for the Afshar hospital and 50.17% for the Firouzgar hospital.	21
**HSPSC, 2014, Saudi Arabia, [32]**	Present findings of a baseline assessment of PSC, compare results with regional and international studies, and explore the association between PSC predictors and outcomes, considering respondent characteristicsand facility size	Variety of staff (physicians, nurses, clinical and non-clinical staff, pharmacy and laboratory staff, dietary and radiology staff, supervisors, and hospital managers) considered. Response rate 85.7% reported. The results compared with other studies using HSPSC Arabic version	Only one tertiary care university teaching hospital considered	Teamwork within units and organizational learning/continuous improvement had high positive responses. Staffing, non-punitive response to errors, and communication openness required improvement. A high correlation was indicated between smaller facilities, events reported, and patient safety levels. Overall perception of safety was 65.3%. Frequency of events reported was 59.4%.	34
**HSPSC, 2015, Turkey, [33]**	Investigate nurses’ perceptions of PSC	HSPSC Turkish version used.	Only nurses in one public hospital considered for collecting data	High positive scores for hospital management support and manager/supervisor expectations and actions supported an increase in patient safety. Frequency of event reporting for medical errors had the lowest positive score. Organizational learning/continuous improvement, hospital management support for patient safety, teamwork within units, and supervisor/manager received high positive scores. Hospital handoffs and transitions, non-punitive response to errors, frequency of events reported, and communication openness were poorly rated. Overall perception of safety was 61%. Frequency of events reported was 40%.	30
**HSPSC, 2016, Iran, [34]**	Evaluate the current status of PSC among hospitals in three central Iran provinces	Variety of staff (doctors, nurses, administrative staff, and paramedics) in the teaching hospitals considered for collecting data.	No Response rate reported	Organizational learning was reported as the strongest dimension. Handoffs and transitions had the lowest score. Overall perception of safety was 62.93%. Frequency of event reporting was 55.63%.	21
**HSPSC, 2012, Turkey, [35]**	Assess health personnel perspectives of PSC in a 900-bed university hospital in Ankara, Turkey	Variety of health professionals (doctors, nurses, technicians, secretaries, and other health personnel) considered Using HSPSC Turkish version.	Only one university hospital considered. Response rate was 43%	Teamwork within units had the highest positive feedback. Frequency of events reported had the lowest average. Women nurses formed the majority of respondents, with five years or less in terms of work experience in their respective hospital. Overall perception of patient safety was 55%. Frequency of events reported was 25%.	21
**HSPSC, 2010, Lebanon, [36]**	Conduct a baseline assessment of PSC in Lebanese hospitals	12,250 staff (physicians, nurses, clinical and non-clinical staff, and others) in 68 hospitals considered. The results compared with U.S. HSPSC Arabic version used.	Response rate was 55.56%	Organizational learning/continuous improvement, hospital management support for patient safety, and teamwork within units were the strongest areas. Non-punitive response to errors and staffing received low feedback. Small hospitals and accredited hospitals received higher scores on several composites. Overall perception of safety was 72.5%. Frequency of events reported was 67.9%.	31
**HSPSC, 2013, Japan and Taiwan, [37]**	Clarify the impact of long nurse working hours on PSC in Japan, the U.S., and Chinese Taiwan using HSPSC	14 hospitals in Japan, 884 hospitals in the U.S., 74 hospitals in Taiwan (acute care hospitals) considered. The results compared with U.S.	Only nurses considered for collecting data. Response rate was Japan = 4047 (58.1%) U.S. = 106,710 (37.0%) Taiwan = 5714 (56.3%)	Patient safety levels declined and number of events reported increased as working hours increased Among the 12 sub-dimensions of PSC, teamwork within units and staffing received poor ratings	29
**HSPSC, 2013, Japan and Taiwan, [38]**	Investigate the characteristics of PSC in Japan, Taiwan, and the U.S.	Variety of health professionals (nurses; patient care assistants/hospital aides/care partners; physicians; pharmacists; dieticians; unit assistants/clerks/secretaries; respiratory therapists; physical, occupational, or speech therapists; technicians (EKG, lab, radiology); administration/management) in 14 hospitals in Japan, 884 hospitals in the U.S., 74 hospitals in Taiwan (acute care hospitals) The results compared with U.S.	Response rate in U.S. = 35.2%	The U.S. had the highest overall positive perception of patient safety grade. Continuous improvement in Japan and the reporting of near-miss events in Taiwan received low scores compared with the other countries. Overall perceptions of patient safety in Japan, the U.S., and Taiwan were 53%, 63%, and 52%, respectively. Frequency of events reported in Japan, the U.S., and Taiwan was 68%, 61%, and 33%, respectively.	30
**SAQ, 2015, India, [39]**	Explore composite patient safety climate, assess various dimensions of patient safety climate in three hospitals, and identify future directions for developing a strong safety climate	Variety of health professionals (clinicians, postgraduates, residents, nurses, and paramedical workers) considered. Response rate was 100%	Only three tertiary care hospitals considered	The study hospitals did not have disparities in the patient safety index score. Different categories of medical workers reported different levels for the perception of management and stress recognition and teamwork. A high correlation exists for perception of management and teamwork with the patient safety index score.	28
**HSPSC, 2017, Sweden, [40]**	Investigate the PSC in all Swedish hospitals; compare the culture among managers, physicians, registered nurses, and enrolled nurses; and identify factors associated with high overall patient safety	Variety of staff (managers, registered nurses, enrolled nurses, and physicians) considered	Only three work areas: general wards, emergency care, and psychiatry care considered. Response rate was 47.4%	Teamwork within units had the most positive feedback. Management support for patient safety received the lowest score. Managers had the highest score for patient safety. Registered nurses had the lowest score for patient safety. Emergency care units showed more patient safety than general wards. Overall perception of patient safety was 58%. Frequency of events reported was 54.4%.	30
**HSPSC, 2013 Netherlands, [41]**	Examine similarities and differences in hospital PSC in three countries: the Netherlands, the U.S., and Taiwan	Variety of staff (nursing staff, medical staff, management and administrative staff, other) in 45 hospitals in the Netherlands, 622 in the U.S., and 74 in Taiwan (non-teaching and teaching hospitals) considered. Comparing the results with U.S., and Taiwan	U.S. Response rate was 52%	Handoffs and transitions required improvement in all three countries. Respondents in U.S. hospitals reported higher levels of PSC than the Taiwanese and Dutch. Differences in responses were evident in hospitals in each country. Overall perceptions of patient safety in the Netherlands, Taiwan, and the U.S. were 49%, 52%, and 64%, respectively. Frequency of events reported in the Netherlands, Taiwan, and the U.S. were 36%, 31%, and 60%, respectively.	24
**HSPSC, 2017, Pakistan, [42]**	Present descriptive statistics for patient safety standards		Only two public hospitals considered. Response rate was 38.4%	80% of respondents indicated there was no response to reported errors in their wards. For respondents that reported errors, an accusatory culture existed in the ward. 70% of respondents reported a lack of support. Feedback from respondents indicated that error reporting and patient safety standards were not favorable.	21
**HSPSC, 201,, Japan, [43]**	Examine the validity and applicability of the HSPSC in Japan and compare the factor structure to the original U.S. study	Variety of healthcare workers (nurses, administrative workers, physicians, technicians, dieticians, pharmacists, therapists, janitors, other) in 13 acute care general hospitals (1 university hospital and 12 teaching hospitals) considered. HSPSC Japanese version used.		The AHRQ’s 12-factor model provides the best fit to the Japanese HSPSC data for acute care hospital staff compared with two 11-factor models proposed in previous studies. The Japanese HSPSC had acceptable internal consistency for the subscales.	31
**HSPSC, 2013, Croatia, [44]**	Determine whether all 12 dimensions of the U.S. HSPSC are applicable, valid, and reliable for Croatian healthcare workers	Considering variety of healthcare workers (doctors and nurses). comparing the results with U.S	Only four Croatian hospitals considered. Response rate was 32.69%	Organizational learning/continuous improvement and staffing had low positive feedback. Confirmatory factor analysis confirmed a good fit to the original U.S. model. Overall perception of patient safety was 57%.	33
**HSPSC, 2013, Sri Lanka, [45]**	Assess the current PSC in a tertiary care hospital	Considering variety of healthcare workers (administrators, consultants, postgraduate trainees, medical officers, house officers, and nursing officers)	Considering only one tertiary care hospital. No Response rate reported	Organizational learning/continuous improvement and teamwork within units had high positive scores. Staffing and workload had low scores. Patient safety overall perception was 81.3%. Frequency of event reporting was 36.3%.	28
**HSPSC, 2012, China, [46]**	Explore nurses’ perceptions of PSC and factors associated with those perceptions		Considering only nurses in one university teaching hospital. No Response ratereported	Organizational learning/continuous improvement and teamwork within units had the highest scores. Low response rates were evident in perceived trustworthiness of managers, non-punitive response to errors, managers, organizational safety prioritization, managers’ safety commitment, and nurses’ years of experience in their units, which had strong correlations with PSC Overall percentage of positive responses regarding patient safety culture was 61.3%.	30
**HSPSC, 2013, China, [47]**	Explore the attitudes and perceptions of PSC for healthcare workers in China and compare the psychometric properties of an adapted translation of the HSPSC in Chinese hospitals with those of the U.S.	Considering variety of health professionals (physicians [surgical clinicians and internal clinicians] and nurses in 32 hospitals. Comparing the results with U.S. HSPSC Chinese version used.		The staffing dimension had the lowest score. Organizational learning/continuous development and teamwork within units had the highest scores. Overall perception of patient safety was 55%.	30
**HSPSC, 2013, Slovenia, [48]**	Study the psychometric properties of a translated version of the HSPSC in a Slovenian setting	Considering variety of health professionals (clinical and non-clinical staff) Comparing the results with other studies HSPSC Slovene version used.	Considering only three acute general hospitals. Response rate was 55%	Units had a greater positive patient safety perception compared with hospital level. The dimensions of teamwork across units, hospital management support for patient safety, staffing, and non-punitive response to errors required improvement. Overall perception of safety was 56%. Frequency of events reported was 69%.	28
**HSPSC, 2010, Belgium, [49]**	Describe a PSC improvement approach in five Belgian hospitals	3940 and 3626 staff (nurses, head nurses, nurse assistants, physicians, head physicians, junior physicians, pharmacists, pharmacy assistants, middle management, technicians, paramedical staff, other) considered. Response rates were 77% and 68%.	Five Belgian acute hospitals (three private hospitals and one public hospital)	Hospital management support for patient safety needed the most improvement. Progress was observed for supervisor expectations and actions promoting safety. Teamwork within units had the highest scores. Staffing, non-punitive response to errors, and hospital transfers and transitions received the lowest scores and did not show signs of improvement.	31
**HSPSC, 2017, Norway, [50]**	Explore organizational factors influencing patient safety and safety behavior among nurses and other hospital staff	Considering 3475 health professionals [nurses (n = 750), other personnel (n = 953)] Studying PSC relationships with safety behavior. HSPSC Norwegian version used.	Considering only one university hospital. Response rate was 49%	Higher values on hospital-level dimensions positively influenced safety leadership and safety climate at the unit level. The organizational factors correlate with the dimensions and illustrate structural relationships that are relevant for variations in the perception of patient safety and safety behavior.	34
**HSPSC, 2010, Taiwan, [51]**	Assess the PSC in Taiwan and attempt to provide an explanation for some of the phenomena that are unique in Taiwan	Considering 1000 health professionals (physicians, nurses, and non-clinical staff) 42 teaching hospitals. Response rate was 78.8% Comparing the results with U.S. HSPSC Chinese version used.		Staffing had the lowest positive feedback. Teamwork within units had the highest score. Statistical examination presented differences between the U.S. and Taiwan in the dimensions of frequency of event reporting, feedback and communication about errors, and communication openness. Overall perception of safety was 65%. Frequency of event reporting was 57%.	35
**HSPSC, 2010, U.S., [52]**	Examine the multilevel psychometric properties of the survey	Considering variety of staff (clinical and non-clinical) in 331 U.S. hospitals. Examine the validity and reliability of the instruments.	Response rate was 55%	Overall, the survey items and dimensions are psychometrically sound at the individual, unit, and hospital levels of analysis and can be used by researchers and hospitals for assessing PSC. A strong correlation existed between patient safety grade and overall perceptions of patient safety and management support for patient safety. Correlation between frequency of event reporting and non-punitive response to errors was poor.	33
**MSI-2006, 2015, Canada, [53]**	Examine in detail how ease of reporting, unit norms of openness, and participative leadership influence front-line staff perceptions of PSC within healthcare organizations	Considering variety of health professionals (nurses, physicians, and pharmacists) in 13 hospitals. Response rate was 17%. Studying PSC relationships with using different outcomes.		Staff perception of patient safety climate was positively correlated to participative leadership, ease of reporting, and unit norms of openness. Demographic factors such as education level and age influenced perceptions of patient safety climate.	35
**SHSQ, 2013, Scotland, [54]**	Obtain a measure of hospital safety climate from a sample of National Health Service (NHS) acute hospitals in Scotland and determine whether these scores are associated with worker safety behaviors and patient and worker injuries	Considering 8113 NHS clinical staff. Examining the validity and reliability of the instruments. Studying PSC relationships with using different outcomes.	Considering only six acute hospitals in Scotland. Response rate was 23%	Patient and worker injury measures and workers’ safety behavior had a significant influence on hospital safety climate scores. Generic safety climate items and patient-specific items had strong impacts on safety outcome measures. Overall perception of safety was 56%. Frequency of incident reporting was 56%.	27
**HSPSC, 2018, Philippines, [55]**	Assess PSC among nurses at a government hospital	Response rate was 86.65%.	Only nurses in one tertiary government hospital considered	Organizational learning and teamwork within units received the highest scores. Non-punitive response to errors had the lowest positive feedback. Overall perception of safety was 50.78%. Frequency of events reported was 54.12%.	29
**HSPSC, 2011, Italy, [56]**	Determine the level of awareness regarding PSC among health professionals working at a hospital in northern Italy	Respondents consisting of five professional groups (directors/coordinators, physicians, nurses/midwives, physiotherapists, and technicians). HSPSC Italian version used.	Only one hospital in northern Italy considered.	Teamwork within units and organizational learning/continuous improvement received the highest scores. Non-punitive response to errors received the lowest score. Overall perception of patient safety was 64%. Frequency of event reporting was 59%.	22
**HSPSC, 2018, South Korea [57]**	Investigate the relationships between registered nurses’ perceptions of PSC in their workplace and their patient safety competency—attitudes, skills, and knowledge	Response rate was 79.7%. Studying PSC relationships with using different outcomes their workplace and their patient safety competency—attitudes, skills, and knowledge. Using HSPSC Korean version and the Patient Safety Competency Self-Evaluation (PSCSE)	Considering only nurses in in one university hospital	A strong correlation existed between teamwork within units and overall safety competency. Attitudes had a strong correlation to teamwork across and within units, and supervisor or manager expectations. Skills had a strong correlation to learning and teamwork within units. Knowledge had a strong correlation to organizational learning.	28
**HSPSC, 2013, Finland, [58]**	Explore and compare nurse managers’ s’ and registered nurses views on PSC to discover whether there are differences between their views	HSPSC Finnish version used.	Considering only nurses in four acute care hospitals. Response rate was 17%	A lack of feedback was evidenced by reporting and communication errors. Expectations and actions of nurse managers at the unit level supporting patient safety had the best positive response from both groups of respondents. Nurse managers at the unit level considered suggestions from staff on how to improve patient safety. Feedback from the survey indicated inadequate hospital-level management support for patient safety.	27
**HSPSC, 2018, India, [59]**	Assess the perceptions of PSC among healthcare providers at a public sector tertiary care hospital in South India	Considering variety of health professionals (doctors, nurses, other technical staff, pharmacists, lab technicians, dialysis technicians, operation theater technicians, and dressing technicians). Response rate was 91.7%	Considering only one tertiary government hospital	Organizational learning/continuous improvement, teamwork within units, and supervisor or officer-in-charge expectations received the highest positive responses while handoffs and transitions, communication openness, and frequency of event reporting received the lowest scores. Overall general perception was 60.8%. Frequency of events reported was 41.2%. Overall general perception among doctors, nurses, and technical staff was 51.6%, 52.8%, and 66.1%, respectively. Frequency of events reported among doctors, nurses, and technical staff was 31.5%, 36.7%, and 46%, respectively.	28
**HSPSC, 2017, China, [60]**	Use the HSPSC to survey PSC in a county hospital in Beijing to determine the strengths and weaknesses of PSC in this hospital	Considering variety of staff (physicians, nurses, and allied health professionals). HSPSC Chinese version used.	Considering only one county hospital.	Frequency of event reporting, communication openness, staffing, and overall perception of patient safety needed potential improvement. Teamwork across units received a high level of positive feedback. Physicians indicated low scores for the majority of the dimensions. Overall perception of safety was 45.0%. Frequency of event reporting was 43.0%.	30
**PSCHO, 2015, China, [61]**	Describe staff’s perceptions of PSC in public hospitals and determine how perceptions of PSC differ between different types of workers in the U.S. and China	Considering variety of staff (managers in administrative offices and clinical departments, non-management physicians, non-management nurses, and others, including medical technicians and others with non-management positions) in six secondary, general public hospitals		Overall perception of patient safety was positive for most dimensions. Hospital managers in both China and the U.S. reported a better patient safety climate than other staff. Scales of fear of shame and blame had the highest response for hospital workers in China. Fear of shame received the lowest feedback among hospital workers in the U.S.	26
**HSPSC, 2014, Portugal, [62]**	Determine the validity and reliability of the AHRQ Hospital Survey on Patient Safety Culture (HSPSC) Portuguese version	Considering variety of hospital staff. HSPSC Portuguese version used.	Response rate was 21.8%	Non-punitive response to errors, management support for patient safety, and staffing had the lowest positive scores. Teamwork within units had the highest score. Overall perception of patient safety was 54%. Frequency of events reported was 40%.	24
**HSPSC, 2014, Jordan, [63]**	Examine the impact of patient safety educational interventions among senior nurses on their perceptions of safety culture and the rate of reported adverse events, pressure ulcers, and patient falls	Studying PSC relationships with using patient safety educational interventions	Considering only nurses in one specialized hospital. Response rate was 57%	Improvements identified by senior nurses included non-punitive response to errors and frequency of event reporting. A reduction in the rate of adverse effects was noted. Pre-education perceptions of safety stood at 51.5% while the post-education perception stood at 60.6%. Frequency of event reporting was 54.2% pre-education and 64.3% post-education.	34
**HSPSC, 2015, Jordan, [64]**	Examine nurses’ perceptions of the hospital safety culture in Jordan and identify the relationships between aspects of hospital safety culture and selected safety outcomes		Considering only nurses in five Jordanian hospitals. Response rate was 61%	Teamwork within units received the highest response Staffing and non-punitive response to errors had the lowest scores Overall perception of patient safety was 43.3% Frequency of event reporting was 37%	30
**SAQ, 2015, Denmark, [65]**	Describe and analyze the patient safety climate in 15 Danish hospital units	Considering variety of staff (doctors, nurses, nursing assist- ants/similar, physiotherapists, occupational therapists, administrative staff, and hospital porters)	Considering only five hospitals	No differences in positive percentage rates were found between nurses and doctors across age, gender, or work experience. Significant differences were noted between front-line staff and leaders. Individuals within a given unit had varied perceptions compared to units within the hospital.	26
**HSPSC, 2015, Belgium, [66]**	Measure differences in safety culture perceptions within Belgian acute hospitals and examine variability based on language, work area, staff position, and work experience	Considering variety of staff (nurses; patient care assistants/hospital aides/care partners; physicians; pharmacists; dieticians; unit assistants/clerks/secretaries; respiratory therapists; physical, occupational, or speech therapists; technicians [EKG, lab, radiology], administration/management) in 89 acute Dutch- and French-speaking hospitals. Studying PSC relationships with using different outcomes using HSPSC Belgian version	Response rate was 51.7%	Staffing, handoffs and transitions, and management support for patient safety were noted as significant problem areas. Overall, Dutch-speaking hospitals had more positive perceptions of PSC than French-speaking hospitals. Respondents working in rehabilitation, pediatrics, and psychiatry gave more positive feedback on PSC. Staffs working in the emergency department, multiple hospital units, and operating theater had lower positive feedback.	30
**HSPSC, 2019, Algeria, [67]**	Measure safety culture dimensions in order to improve and promote healthcare in Algeria	Considering variety of staff (nursing assistants, nurses, doctors, administrative staff, other)	Considering only one General hospital. No Response rate reported	Organization learning/continuous learning and teamwork within units had the highest scores. Communication openness and staffing had the lowest scores. Overall patient safety perception was 76.3%. Frequency of events reported was 56.1%.	25
**HSPSC, 2009, U.S., [68]**	Analyze the psychometric properties of the Agency for Healthcare Research and Quality Hospital Survey on Patient Safety Culture (HSPSC)	Considering variety of staff (included nurses, physicians, pharmacists, and other hospital staff members) Response rate was 96%. Examining the validity and reliability of the instruments.	Only three hospitals (an academic teaching hospital, a managed care organization hospital, and a private not-for-profit community hospital) considered	Interitem consistency reliability was not less than 0.7 for 5 subscales; the least reliability coefficients were demonstrated by the staffing subscale. The intraclass correlation coefficients were within normal ranges. Similar patterns of high and low scores across the subscales of the HSPSC were noted and compared to the sample from Pacific region hospitals conveyed by the Agency for Healthcare Research and Quality and corresponded to the proportion of items in each subscale that are reverse scored. Most of the unit and hospital dimensions revealed a positive relationship with the Safety Grade outcome measure.	32
**HSPSC and SAQ, 2012, U.S., [69]**	Examine the reliability and predictive validity of two patient safety culture surveys- Safety Attitudes Questionnaire (SAQ) and Hospital Survey on Patient Safety Culture (HSPSC)-when administered to the same participants. Additionally, to determine the ability to convert HSOPS scores to SAQ scores.	Variety of non-physician employees considered. Examining the validity and reliability of the instruments using HSPSC and SAQ. Considering intensive care units (ICUs) in 12 hospitals within a large hospital system in the southern United States	Response rate was 54%. Only non-physician employees considered.	Frequency of event reporting, perception of general patient safety, and general patient safety grade had a significant relationship with SAQ and HSPSC at individual level, with correlations of r = 0.41 to 0.65 for SAQ and from r = 0.22 to 0.72 for HSOPS. Neither SAQ nor HSPSC predicted the fourth HSOPS outcome, i.e., the number of events reported within the last year. Analyses on regression revealed that HSPSC safety culture dimensions had the best ability to predict frequency of event reporting and general perceptions of patient safety while SAQ and HSPSC dimensions predicted patient safety grade only.	34
**HSPSC, 2010, U.S., [70]**	Examine relationships between the Agency for Healthcare Research and Quality’s (AHRQ) Hospital Survey of Patient Safety Culture and rates of in-hospital complications and adverse events as measured by the AHRQ Patient Safety Indicators (PSIs)	56,480 staff from 179 hospitals considered. Studying PSC relationships with using PSI data.		Exploratory analysis done showed that hospitals which scored higher on patient safety culture had fewer reported adverse events, after controlling for hospital bed size, teaching status, and ownership. There was a significant correlation between hospital bed size, teaching status, and ownership and the PSI composite. Larger and private hospitals had higher PSI rates. Almost all tested relationships were aligning to the hypothesis (negative), and 7 of the 15 relationships were statistically significant and HSPSC composite average (47%). All significant relationships had standardized regression coefficients between −0.15 and −0.41, denoting that hospitals with higher positive PSC scores experienced less in-hospital complications/adverse events as measured by PSIs.	28
**HSPSC, 2016, U.S., [71]**	Analyze how different elements of patient safety culture is associated with clinical handoffs and perceptions of patient safety	885 hospitals considered for collecting data		Positive patient safety perceptions were influenced by effective information handoff, responsibility, and accountability. There was positive correlation between feedback and communication of errors and conveying of patient information. Teamwork within units and the frequency of events documented had positive correlation to the transference of personal responsibility when changing shifts.	35
**HSPSC, 2009, U.S., [72]**	Investigate the existence of a patient safety chain for hospitals	371 hospitals considered	Response rate was 59.3%.	TFL has a role in creating a PSC through the actual PSI execution. TFL has an indirect relationship with the implementation of initiatives, and ultimately improved PSO. The characteristics of inspirational leaders are linked with the creation and promotion of a safety culture, making safety a priority and investing resources to PSI to realize maximal improvements in PSO.	26
**SAQ, 2006, U.S., UK, and NZ, [73]**	Describe the survey’s background, psychometric characteristics, provide benchmarking data, discuss how the survey can be used, and note emerging areas of research	203 sites were considered. Examining the validity and reliability of the instruments.		A six-factor model used at both the clinical area and respondent nested within clinical area levels generated attitudes. The factors were: Teamwork Climate, Safety Climate, Perceptions of Management, Job Satisfaction, Working Conditions, and Stress Recognition. With a scale reliability of 0.9, provider attitudes varied significantly within and among organizations. Using SAQ to measure climate in clinical areas permits comparisons between hospitals, patient care areas, and types of caregivers, and tracking of change over time.	30
**PSCHO, 2007, U.S., [74]**	Describe the development of an instrument for assessing workforce perceptions of hospital safety culture and to assess its reliability and validity	100 Hospitals considered. Examining the validity and reliability of the instruments	response rate was 51%	Nine constructs were acknowledged: three organizational factors, two unit factors, three individual factors, and one additional factor. Constructs showed significant convergent and discriminant validity in the MTA. Cronbach’s a coefficient ranged from 0.50 to 0.89.	29
**PSCHO, 2009, U.S., [75]**	Examine the relationship between measures of hospital safety climate and hospital performance on selected Patient Safety Indicators (PSIs).	91 hospitals considered. Examining the validity and reliability of the instruments. Studying PSC relationships with PSIs.	Response rate was 52%.	Hospitals showing better safety climate had lower relative incidence of PSIs. Those with higher scores on safety climate dimensions determining interpersonal beliefs regarding shame and blame. Frontline worker’s perceived lower risk of experiencing PSIs related to better safety climate, however, senior managers did not agree on this.	31
**PSCHO, 2011, U.S., [76]**	Define the relationship between hospital patient safety climate (a measure of hospitals’ organizational culture as related to patient safety) and hospitals’ rates of rehospitalization within 30 days of discharge	67 hospitals considered. Examining the validity and reliability of the instruments. Studying PSC relationships with rates of rehospitalization.	Response rate was 38.5 %	There was a noteworthy positive correlation between lower safety climate and higher rates of readmission among AMI (acute myocardial infarction) and HF (heart failure) (p 0.05 for both models). Frontline workers perceptions of safety climate were linked to readmission rates (*p* 0.01), however, the management’s perceptions contradicted this. The results demonstrate that hospital patient safety climate has a connection with readmission outcomes patients with AMI and HF. The associations were specific to management and leadership.	24
**PSCHO, 2008, U.S., [77]**	Determine whether frontline workers and supervisors perceive a more negative patient safety climate than senior managers in their institutions.	92 US Hospitals considered. Examining the validity and reliability of the instruments.		Frontline personnel’s safety climate perceptions were 4.8, percentage points (1.4 times) more problematic than senior managers’, and supervisors’ perceptions were 3.1 percentage points (1.25 times) more problematic than senior managers’. Discipline had an impact on the differences at management level: senior managers had less differences than frontline workers. Additionally, the differences were more pronounced for nurses than physicians and other disciplines.	34

**Table 3:** Five measurements of PSC dimensions.

Survey	PSC Dimensions
HSPSC	Management support for PS Teamwork within units Teamwork across units Communication openness Frequency of events reported Feedback and communication about errors Organizational learning—continuous improvement Nonpunitive responses to errors Handoffs and transitions Staffing Supervisor/manager expectations and actions that promote PS Overall perceptions of PS
SAQ	Teamwork climate Safety climate Job satisfaction Stress recognition Perceptions of management Working conditions
PSCHO	Engagement of senior managers Organizational resources Overall emphasis on PS Unit safety norms Unit support and recognition for safety efforts Fear of blame Fear of shame
MSI	Organization leadership for safety Unit leadership for safety Perceived state of safety Shame and repercussions of reporting Safety learning behaviors
SHSQ	Supervisors’ expectations and actions Organizational learning/improvement Teamwork within hospital units Communication openness Feedback and communication about error Non-punitive responses to errors Staffing Hospital management support for PS Teamwork across hospital units Hospital handoffs Frequency of incident reporting Overall perceptions of safety

## Data Availability

Not relevant to this study.
